# Acceptability of a herd immunity-focused, transmission-blocking malaria vaccine in malaria-endemic communities in the Peruvian Amazon: an exploratory study

**DOI:** 10.1186/s12936-018-2328-z

**Published:** 2018-04-27

**Authors:** Sara E. White, Steven A. Harvey, Graciela Meza, Alejandro Llanos, Mitchel Guzman, Dionicia Gamboa, Joseph M. Vinetz

**Affiliations:** 1 Division of Infectious Diseases, Department of Medicine, University of California, San Diego School of Medicine, 9500 Gilman Drive 0760, Biomedical Research Facility Room 4A16, La Jolla, CA 92093-0760 USA; 2 Department of International Health, Johns Hopkins Bloomberg School of Public Health, 615 N. Wolfe St. E5030, Baltimore, MD 21205 USA; 3 Facultad de Medicina Humana, Universidad Nacional de la Amazonia Peruana, Iquitos, Peru; 4 Malaria and Leishmaniasis Division, Instituto de Medicina Tropical Alexander von Humboldt, Av. Honorio Delgado 430, San Martín de Porres, Lima, Peru; 5 Department of Cellular and Molecular Sciences, Faculty of Sciences and Laboratory of Research and Development, Universidad Peruana Cayetano Heredia, Lima, Peru

**Keywords:** Malaria, Transmission-blocking vaccine (TBV), Social acceptability, Peru, Amazon

## Abstract

**Background:**

A transmission-blocking vaccine (TBV) to prevent malaria-infected humans from infecting mosquitoes has been increasingly considered as a tool for malaria control and elimination. This study tested the hypothesis that a malaria TBV would be acceptable among residents of a malaria-hypoendemic region.

**Methods:**

The study was carried out in six Spanish-speaking rural villages in the Department of Loreto in the Peruvian Amazon. These villages comprise a cohort of 430 households associated with the Peru-Brazil International Centre for Excellence in Malaria Research. Individuals from one-third (143) of enrolled households in an ongoing longitudinal, prospective cohort study in 6 communities in Loreto, Peru, were randomly selected to participate by answering a pre-validated questionnaire.

**Results:**

All 143 participants expressed desire for a malaria vaccine in general; only 1 (0.7%) expressed unwillingness to receive a transmission-blocking malaria vaccine. Injection was considered most acceptable for adults (97.2%); for children drops in the mouth were preferred (96.8%). Acceptability waned marginally with the prospect of multiple injections (83.8%) and different projected efficacies at 70 and 50% (90.1 and 71.8%, respectively). Respondents demonstrated clear understanding that the vaccine was for community, rather than personal, protection against malaria infection.

**Discussion:**

In this setting of the Peruvian Amazon, a transmission-blocking malaria vaccine was found to be almost universally acceptable. This study is the first to report that residents of a malaria-endemic region have been queried regarding a malaria vaccine strategy that policy-makers in the industrialized world often dismiss as altruistic.

**Electronic supplementary material:**

The online version of this article (10.1186/s12936-018-2328-z) contains supplementary material, which is available to authorized users.

## Background

Proof of concept of a malaria transmission-blocking vaccine (TBV) to block the transmission of malaria from human host to mosquito vector was first demonstrated in two landmark studies in the 1970s [1, 2]. From almost the very start, the essential concept of a TBV has been considered—and sometimes dismissed as—altruistic, or only as adjunct to an anti-infection or anti-disease vaccine [3, 4]. A recent review, however, noted the compelling ethical justification of the potential deployment of a highly safe and effective TBV because malaria control, elimination, and eradication are sufficiently important public health goals [5].

Pfs25, first reported in 1988, is the leading antigen candidate for a TBV [6–20], although other candidates are also under development [21]. A purely transmission-blocking vaccine would not directly confer protection against malarial disease to the recipient. Instead, it would prevent infection of the vector by stimulating antibody production against mosquito-infective forms, such as gametocytes, zygotes, and ookinetes. Antibodies would be taken up along with an infectious blood meal and would inhibit further parasite development within the mosquito. Thus, a TBV would prevent infection of mosquitoes, resulting in reduced transmission at the population level [22–28].

Such a concept seems more relevant today amid the increasingly widespread realization that herd immunity in populations does not need to focus solely on prevention of the recipient from becoming infected. This principle has been cited as an antecedent justification for a malaria TBV based, for example, on vaccines against measles [29], rubella [29], human papilloma virus [30], and polio [4]. Even in proposed HIV vaccination strategies, the public health emphasis includes a clear focus on preventing individuals from spreading infection [31]. Regardless, a key gap remains in developing a malaria TBV: to understand whether malaria-affected populations would be willing to accept a vaccine that conferred no direct personal protection.

The end of indoor residual spraying with DDT in the 1990s was followed by an epidemic rise in malaria in the Peruvian Amazon [32–34]. This was followed in turn by a deceptively promising decline in overall incidence, resulting in a stable state of hypoendemic transmission. Recent years have seen continued hyperendemicity, rises in *Plasmodium vivax* incidence, epidemic *Plasmodium falciparum* outbreaks (Peruvian Ministry of Health, unpublished data), and unexpectedly high levels of subclinical and sub patent cases [35–37]. All this suggests that in the long term, prevailing preventive measures [32, 33] may not be sufficient to control, much less eliminate, malaria in this region [38–40]. For instance, the effectiveness of long-lasting insecticide-impregnated bed nets (LLINs) is potentially limited by mosquito behavioural changes in response to net use [41], despite data suggesting that Amazonian vectors remain susceptible to standard insecticides in the region [42]. Test-and-treat strategies only identify clinical cases of malaria, but do not eliminate the asymptomatic reservoir of infection [43]. Other malaria-endemic areas in Amazonia [36, 44] and around the world have seen similar trends [45–47]. It is widely agreed that an effective malaria vaccine would be an important tool to contribute towards regional and global malaria control and elimination [32, 33, 37, 48–50].

TBV development to date has largely assumed that the focus—manifested most recently as the target product profile—would be on preventing infected individuals from infecting vectors in proximity to their local family members or close neighbours where there is anophelism [21]. Recently enhanced insight into how human mobility contributes to maintaining and spreading malaria infections in regions and among populations brings new justification for deploying a TBV, since vaccine recipients with asymptomatic parasitaemia would be prevented from transmitting their parasites to mosquitoes both in their home villages and when moving or travelling to other places [51–56]. No previous study has been published that measures the attitudes of people living in malaria-endemic regions about the general concept of a TBV. This study tested the hypothesis that people living in malaria-hypoendemic communities in the Peruvian Amazon would be able to understand the concept of, and be willing to receive, a transmission-blocking malaria vaccine for themselves and their children. Data to support such a finding would provide important justification and impetus for further TBV development.

## Methods

### Human subjects approvals

This study was approved by the Comité Institucional de Ética (CIE) of the Universidad Peruana Cayetano Heredia, by the Human Research Protections Program of the University of California, San Diego, and by the Dirección Regional de Salud (DIRESA) of the Regional Government of Loreto. The surveyor explained to each participant that his or her participation was voluntary and confidential, and each signed an informed consent. The surveys do not include any identifying information.

### Study population

The study was carried out among a population of 4000 in six Spanish-speaking rural villages in the Loreto Department of the Peruvian Amazon in northeastern Peru, the general and geographic characteristics of which have been previously described [57, 58]. The prevalence of malaria parasitaemia in this region ranges from 3 to 10% [40]. The entire region of northeastern Peru experiences approximately 60,000 cases of malaria annually [40, 58], with an approximate 5:1 ratio of *P. vivax* to *P. falciparum* [40]. The study communities of San Pedro, Santa Rita, and San José de Lupuna are located along the Nanay River; Cahuide, La Habana, and 12 de Abril are located roughly 56 kilometres Southwest of Iquitos along the Iquitos-Nauta highway. The study cohorts based here are part of the Peru-Brazil International Centre for Excellence in Malaria Research (ICEMR) programme.

### Study design and sample size estimates

The parent malaria cohort from which the present study participants were drawn includes 430 numbered households. For feasibility, a sampling rate of 1/3 was determined, yielding a total of 143 households; a formal sample size calculation was not done because of uncertainty about assumptions and outcomes. Retrospective calculations indicated that a sample size of 204 would have been required to estimate a true population acceptability rate of 50% when applying a finite population correction and permitting a 5% margin of error. Although this would have been the most conservative sample size possible, our sample size of 143 was still sufficiently large to estimate a true population-wide rate of TBV acceptance of 84% or higher with a 5% margin of error. Given this point estimate of 99.3%, the study’s sample size is reasonable.

The initial household was selected at random and every third numbered household after it was identified for participation. The head of household, male or female, answered the questionnaire, with the pair answering jointly if desired. Participants were required to be at least 18 years of age. If, after visiting a house on at least two occasions, the head of household was not present, an adult–child was permitted to answer. None of the families approached for participation refused. However, in approximately 20 cases neither a head of household nor an adult–child was present after two visits, so the subsequent listed household was approached instead. Approximately five of these visits were likewise unsuccessful, so respondents at the household prior to the first were sought. This method always elicited a willing participant; the originally determined sample size of 143 did not change as a result.

### Questionnaire format

A quantitative questionnaire was designed to assess the acceptability of a transmission-blocking malaria vaccine for community members, both adults and children. Questions were also included to evaluate changes in acceptability given differences in vaccine cost and efficacy, mode of administration, and potential need for multiple doses. One open-ended question was included to assess respondents’ rationale for accepting or rejecting a transmission-blocking malaria vaccine.

The questionnaire was pre-validated by 12 members of the community of Cahuide using a qualitative group interview format similar to a focus group. The individual consent form and each question were read to the group and the intended meaning expressed. The group was then given the opportunity to suggest alternative ways of phrasing any ambiguous wording. Their suggestions were integrated into the final version of the questionnaire. Individuals involved in questionnaire validation were not eligible to be selected as respondents during data collection.

The questionnaire included 24 questions and required approximately 10 min to complete. Since many respondents were unable to read or write, the investigator administered the questionnaire orally. A Peruvian ICEMR staff member, already well-known to community residents, was present at every interaction. Respondents provided demographic information, including age, gender, occupation, religion, and education level. Respondents were asked about their and their children’s experience with malaria, current prevention practices, and past vaccination history. Before inquiring about willingness to receive a transmission-blocking malaria vaccine, the questionnaire explained the concept as follows (translated from Spanish):

*‘A traditional vaccine prevents you from contracting the illness. But malaria isn’t a traditional illness because it is transmitted from a person to mosquito and then from that mosquito to another person, not simply from person to person like some other diseases. So imagine that there was a malaria vaccine, but it wouldn’t prevent you from getting malaria. Instead it would prevent the mosquitoes that bite you from getting malaria and transmitting it to other people. In other words, even after getting the vaccine, you could still get sick with malaria, but you would no longer pass it on to others in your community’.*

Respondents were then asked whether they would be willing to receive such a vaccine and have it administered to their children. Finally, queries were made to the respondents whether their willingness would change depending on cost, efficacy, number of doses required, and mode of administration. All participants were free to ask questions at any time or ask for clarification of any question. Because of the pre-validation process the questionnaire was generally well-understood; however, the question regarding percent efficacy occasionally caused confusion among participants and required clarification. The concept was always explained as follows: “70% efficacy means that if you give the vaccine to 100 people, it only works in 70 of them, and they no longer transmit malaria. But in the other 30 people, the vaccine does not work, and they continue to pass on malaria to the mosquitoes that bite them.” The questionnaire is provided in Additional files 1 and 2.

## Results

A total of 143 participants were surveyed among the six communities (Table 1). The majority (62%) were women; male heads of household were frequently working outside of the home when their household was approached. A wide range of ages was represented across all communities, from 18 to 78. Almost all (96%) had children, with 76% still living with children in their homes. Agriculture and homemaking were the most common occupations and Catholicism the predominant religion. The majority (86%) of respondents had no formal education or terminated their studies before completing secondary school.

**Table 1:** Demographics of the study participants

	Cahuide	12 de Abril	La Habana	San José de Lupuna	Santa Rita	San Pedro	Total
Total households surveyed							
	48	17	13	28	22	15	143
Gender							
Men	14	9	7	10	10	5	55
Women	34	8	6	18	12	10	88
Age							
18–30	14	3	4	7	2	3	33
31–40	8	4	3	8	7	2	32
41–50	7	4	2	3	6	3	25
51–60	10	1	1	5	2	2	21
61–70	6	2	3	3	1	4	19
71 +	3	3	0	2	4	1	13
Household characteristics							
Childless	3	0	2	1	0	0	6
Average number of children (range)	4.6 (0–14)	4.8 (1–9)	4.3 (0–9)	3.6 (0–10)	4.4 (2–7)	5 (1–9)	4.4 (0–14)
Households living with children < 18 years	37	12	12	22	15	11	109
Average household size (range)	4.4 (1–9)	5.2 (1–13)	5 (2–9)	4.1 (2–7)	4.4 (1–8)	4.7 (1–12)	4.5 (1–13)
Occupation							
Agriculture	21	10	8	9	14	6	68
Homemaker	19	5	3	14	7	7	55
Carpentry	1	1	0	1	0	0	3
Sales	3	1	0	1	0	0	5
Other	4	0	2	3	1	2	12
Religion							
Catholic	24	11	5	15	10	14	79
Evangelical	14	4	3	9	6	1	37
None	6	1	5	3	1	0	16
Other	4	1	0	1	5	0	11
Education level							
No school	7	1	0	0	0	0	8
Primary incomplete	16	7	6	12	13	6	60
Primary complete	6	3	2	5	3	2	21
Secondary incomplete	12	5	3	6	1	7	34
Secondary complete	7	1	2	3	5	0	18
Some university	0	0	0	2	0	0	2

**Fig. 1:**
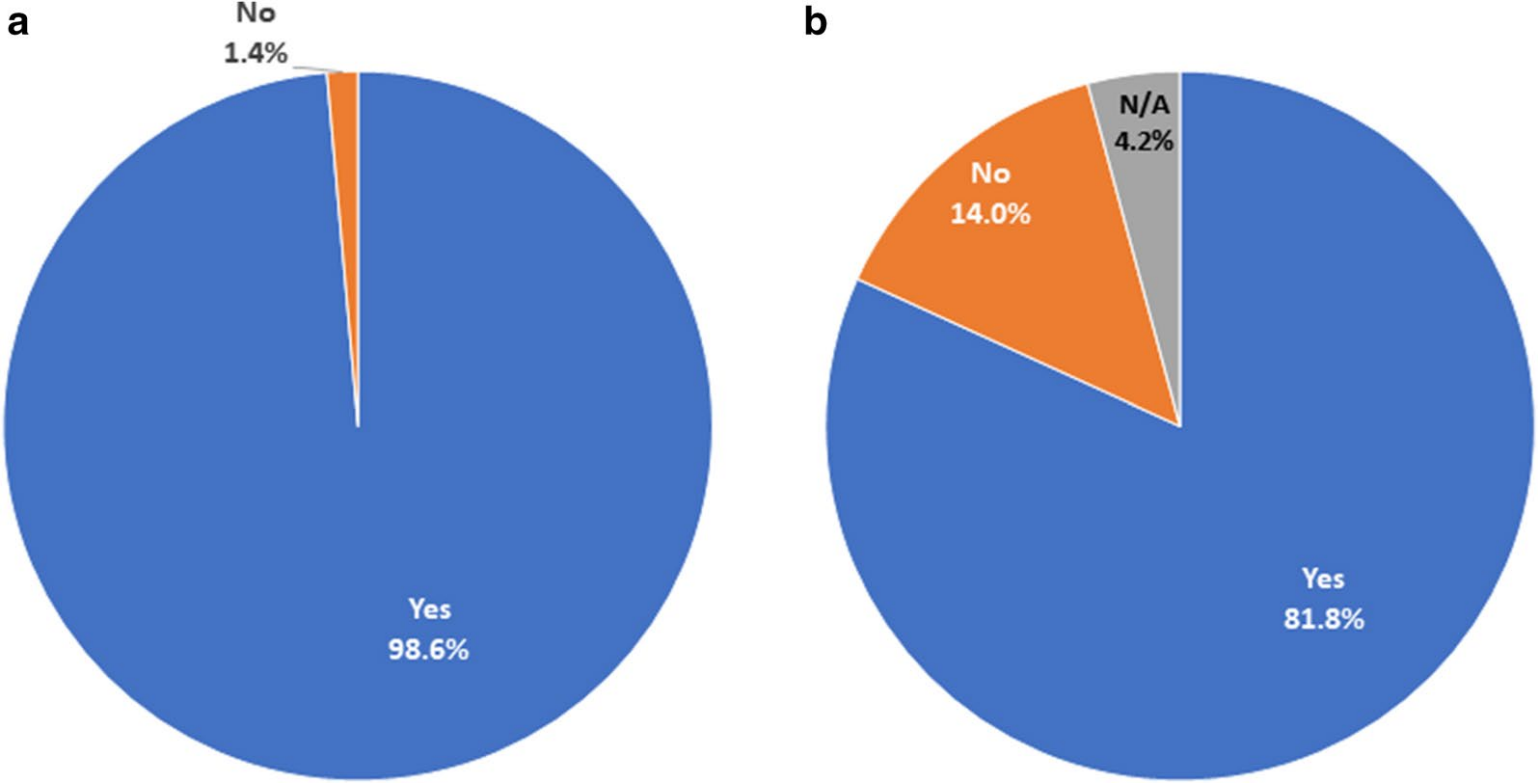
Participants’ answers about whether they (**a**) or any of their children (**b**) have ever had malaria. The number of participants was 143

Participants were asked about their own experience with malaria and that of their children (applicable for 137 respondents). Only 2 participants reported never having malaria (1.4%), and the vast majority (117, 85.4%) reported at least one child who had had malaria (Fig. 1).

**Fig. 2:**
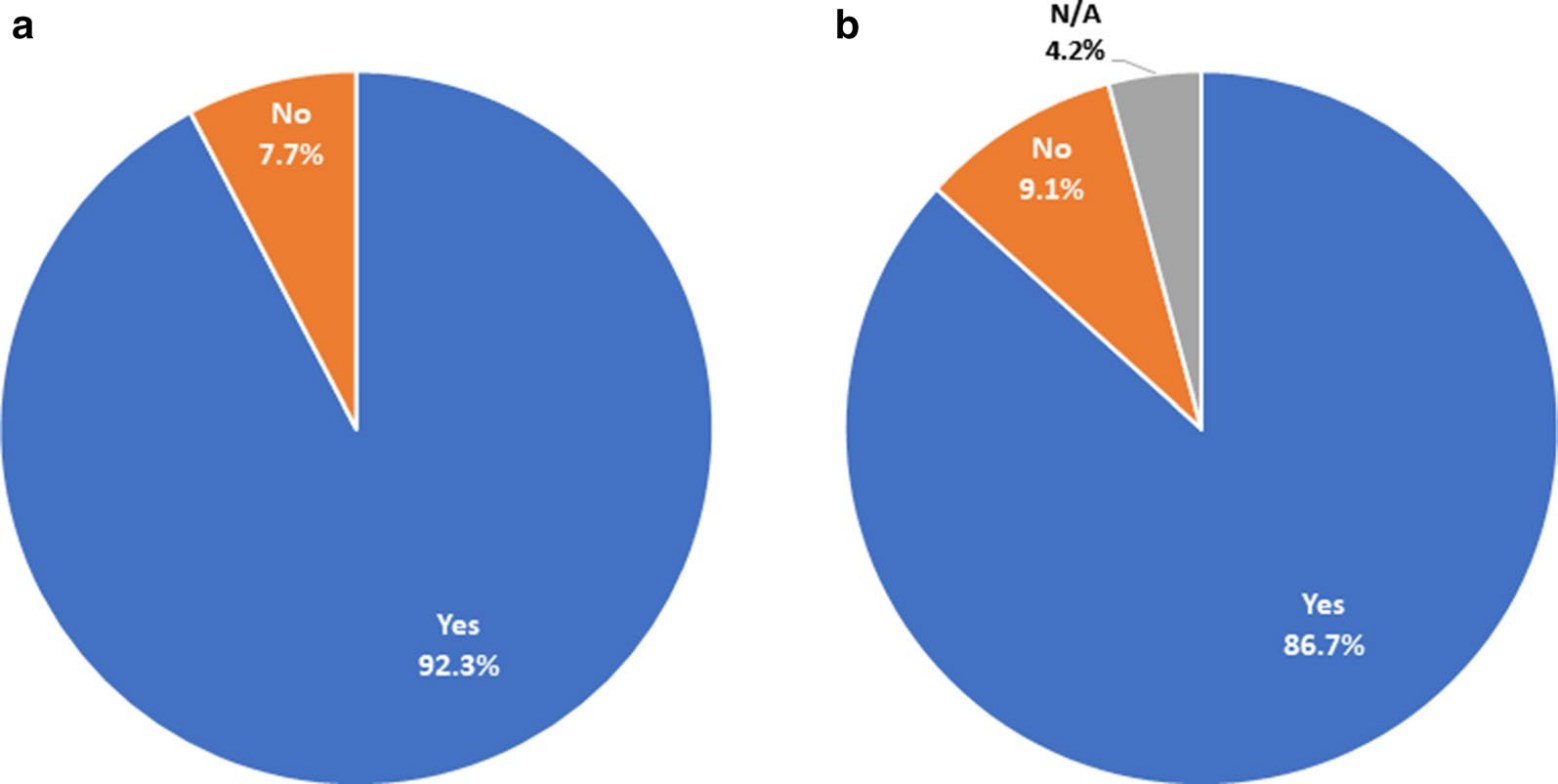
Participants’ answers about whether they (**a**) or any of their children (**b**) have ever received a vaccine. The number of participants was 143

Because acceptability of a malaria vaccine might be influenced by a community’s general culture of vaccine acceptance or non-acceptance, study participants were asked if they and any of their children had ever received any vaccine against another illness and were asked to list those they could remember. Most participants reported that they (131, 92.3%) and their children (124, 90.5%) had received at least one vaccine, with yellow fever, tetanus, varicella, and hepatitis the most commonly cited (Fig. 2).

**Fig. 3:**
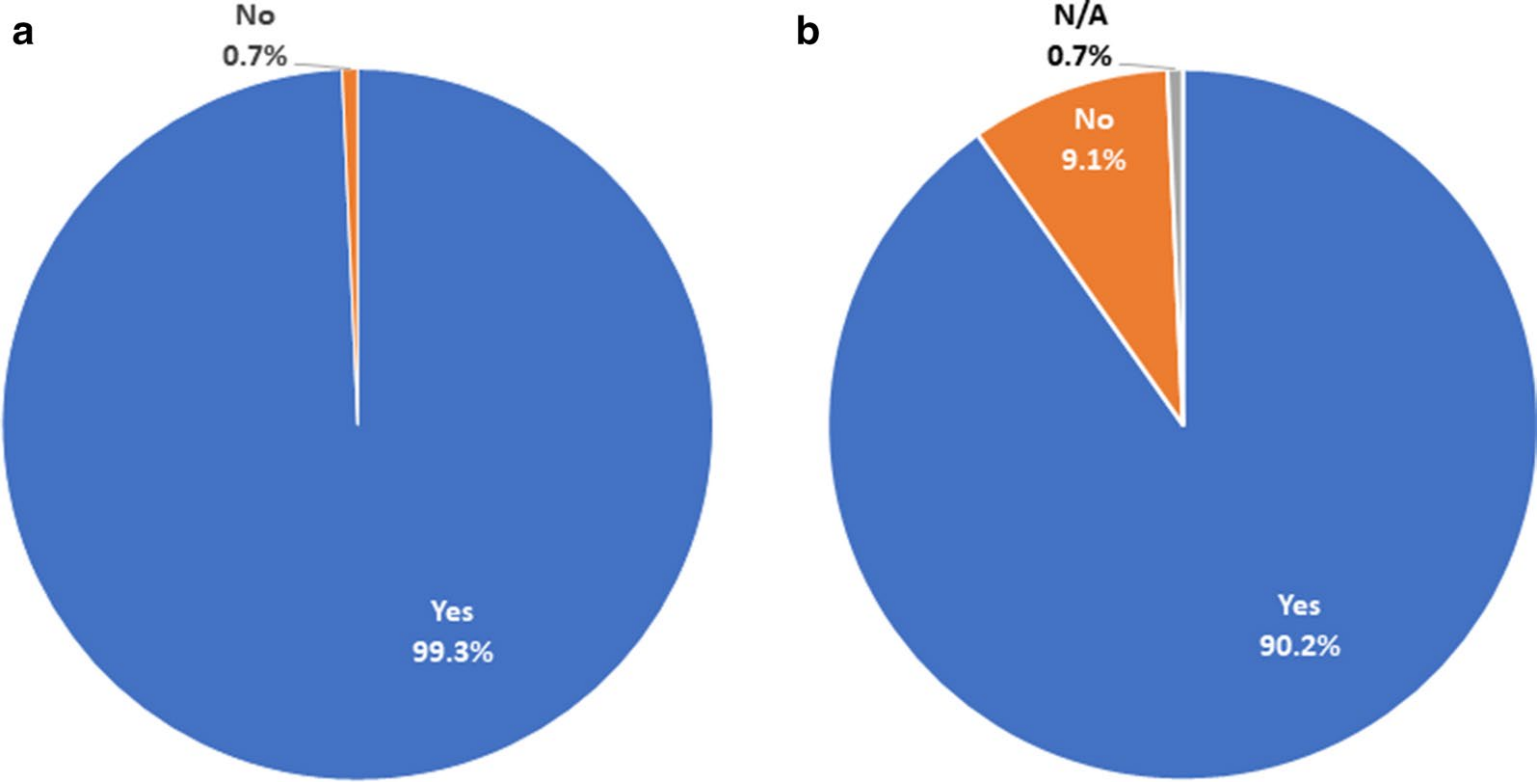
Participants’ answers about whether they would be willing to receive a transmission-blocking malaria vaccine (**a**) and whether they would be willing to give a transmission-blocking malaria vaccine to their children (**b**). The number of participants was 143

Before the mention of a transmission-blocking vaccine, participants were asked whether they desired a malaria vaccine (Fig. 3). All 143 responded that they did. After the explanation of a transmission-blocking vaccine, participants were asked whether they would be willing to receive such a vaccine against malaria. All but one (99.3%) responded that they would. When asked the same question for their children, the same participant was the only one to respond no. All others responded affirmatively (*n* = 124) or said that the question was not applicable either because they had no children or their children were grown (*n* = 18). Despite understanding that the vaccine would provide no protection to them individually, participants nearly universally accepted the idea of a transmission-blocking malaria vaccine for themselves and their children.

**Fig. 4:**
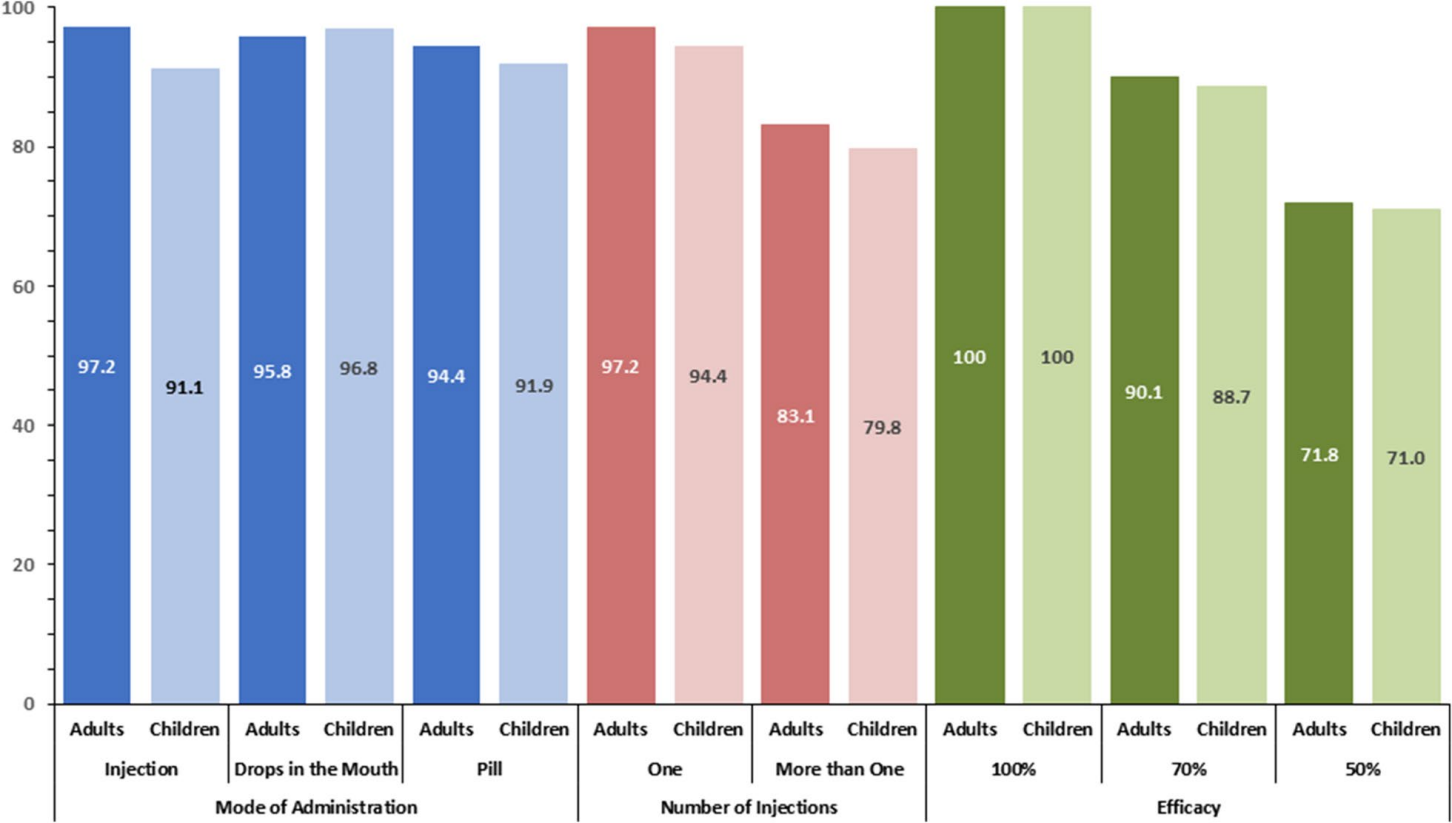
Percent of participants in all 6 surveyed communities who stated that a transmission-blocking malaria vaccine would be acceptable for themselves and their children depending on varying vaccine characteristics. The number of participants was 143

When asked about willingness to pay, 39.0% responded that they would only receive the vaccine if it were free. Sixty-one percent responded that they would be willing to pay a mean price of 6.6 Peruvian soles (about US $2.21 at the time of the study). Acceptability of the vaccine waned marginally depending on mode of administration, with injections being preferred for adults (138/142, 97.2%) and drops in the mouth preferred for children (120/124, 96.8%). When asked about willingness to receive the vaccine if it were necessary to administer multiple injections, reported acceptability decreased to 83.1% for adults and 79.8% for children. When vaccine efficacy was presumed to be 70%, reported acceptability decreased to 90.1% for adults and 88.7% for children; when presumed efficacy was reduced to 50%, reported acceptability decreased to 71.8 and 71.0% for adults and children, respectively (Fig. 4 and Table 2).

**Table 2:** Participants’ willingness to receive a transmission-blocking malaria vaccine depending on varying vaccine characteristics

	Cahuide (*n* = 48)	12 de Abril (*n* = 17)	La Habana (*n* = 13)	San José de Lupuna (*n* = 27)	Santa Rita (*n* = 21)	San Pedro (*n* = 15)	Total (or Average) (*n* = 141)
Cost							
Only if free	22 (45.8%)	3 (17.6%)	4 (30.8%)	11 (40.7%)	8 (38.1%)	7 (46.7%)	55 (39.0%)
Willing to pay	26 (54.2%)	14 (82.4%)	9 (69.2%)	16 (59.3%)	13 (61.9%)	8 (53.3%)	86 (61.0%)
Average price (S./)^a^	8.7	9.4	10.0	2.6	4.3	2.8	6.6
Modified price (S./)^a^	4.7	7.7	6.9	1.5	2.7	1.5	4.0
Price range (S./)^a^	2—30	2—30	5—20	1—5	1—5	1—5	1—5

	Cahuide (*n* = 48)	12 de Abril (*n* = 17)	La Habana (*n* = 13)	San José de Lupuna (*n* = 27)	Santa Rita (*n* = 22)	San Pedro (*n* = 15)	Total (*n* = 142)
Acceptable modes of administration for adults							
Injection	47 (97.9%)	17 (100%)	13 (100%)	27 (100%)	21 (95.5%)	13 (86.7%)	138 (97.2%)
Drops in the mouth	46 (95.8%)	17 (100%)	13 (100%)	25 (92.6%)	21 (95.5%)	14 (93.3%)	136 (95.8%)
Pill	44 (91.7%)	16 (94.1%)	12 (92.3%)	26 (96.3%)	21 (95.5%)	15 (100%)	134 (94.4%)

	Cahuide (*n* = 39)	12 de Abril (*n* = 17)	La Habana (*n* = 11)	San José de Lupuna (*n* = 25)	Santa Rita (*n* = 19)	San Pedro (*n* = 13)	Total (*n* = 124)
Acceptable modes of administration for children							
Injection	34 (87.2%)	16 (94.1%)	10 (90.9%)	23 (92.0%)	18 (94.7%)	12 (92.3%)	113 (91.1%)
Drops in the mouth	37 (94.9%)	17 (100%)	11 (100%)	24 (96.0%)	19 (100%)	12 (92.3%)	120 (96.8%)
Pill	35 (89.7%)	17 (100%)	10 (90.9%)	22 (88.0%)	17 (89.5%)	13 (100%)	114 (91.9%)

	Cahuide (*n* = 48)	12 de Abril (*n* = 17)	La Habana (*n* = 13)	San José de Lupuna (*n* = 27)	Santa Rita (*n* = 22)	San Pedro (*n* = 15)	Total (*n* = 142)
Number of injections acceptable for adults							
One	47 (97.9%)	16 (94.1%)	13 (100%)	27 (100%)	22 (100%)	13 (86.7%)	138 (97.2%)
More than one	38 (79.2%)	14 (82.4%)	13 (100%)	25 (92.6%)	17 (77.3%)	11 (73.3%)	118 (83.1%)

	Cahuide (n = 39)	12 de Abril (n = 17)	La Habana (n = 11)	San José de Lupuna (n = 25)	Santa Rita (n = 19)	San Pedro (n = 13)	Total (n = 124)
Number of injections acceptable for children							
One	35 (89.7%)	17 (100%)	10 (90.9%)	24 (96.0%)	18 (94.7%)	13 (100%)	117 (94.4%)
More than one	28 (71.8%)	15 (88.2%)	10 (90.9%)	22 (88.0%)	13 (68.4%)	11 (84.6%)	99 (79.8%)

	Cahuide (*n* = 48)	12 de Abril (*n* = 17)	La Habana (*n* = 13)	San José de Lupuna (*n* = 27)	Santa Rita (*n* = 22)	San Pedro (*n* = 15)	Total (*n* = 142)
Acceptable efficacy for adults (%)							
100	48 (100%)	17 (100%)	13 (100%)	27 (100%)	22 (100%)	15 (100%)	142 (100%)
70	43 (89.6%)	15 (88.2%)	12 (92.3%)	25 (92.6%)	21 (95.5%)	12 (80.0%)	128 (90.1%)
50	29 (60.4%)	12 (70.6%)	10 (76.9%)	22 (81.5%)	17 (77.3%)	12 (80.0%)	102 (71.8%)

	Cahuide (*n* = 39)	12 de Abril (*n* = 17)	La Habana (*n* = 11)	San José de Lupuna (*n* = 25)	Santa Rita (*n* = 19)	San Pedro (*n* = 13)	Total (*n* = 124)
Acceptable efficacy for children (%)							
100	39 (100%)	17 (100%)	11 (100%)	25 (100%)	19 (100%)	13 (100%)	124 (100%)
70	35 (89.7%)	15 (88.2%)	10 (90.9%)	23 (92.0%)	17 (89.5%)	10 (76.9%)	110 (88.7%)
50	22 (56.4%)	12 (70.6%)	8 (72.7%)	21 (84.0%)	15 (78.9%)	10 (76.9%)	88 (71.0%)

^a^Prices are listed in Peruvian soles. In July–August 2014, S./1 ≈ $0.36 USD. Average price is the mean of the prices cited by those who were willing to pay for the vaccine. Modified price is the mean of all prices, including the price of S./0 cited by those who were only willing to receive the vaccine if it were free. Six participants were childless and 12 responded that they did not wish to respond on behalf of their adult children who could make their own vaccination decisions, accounting for n = 142 for adult responses and n = 124 for responses related to children

Only one of 143 participants in the study considered the rationale for a transmission-blocking malaria vaccine to be unacceptable; unfortunately, information was not available regarding why she responded negatively. This respondent was a homemaker and mother living in one of the Nanay River Basin communities. Unlike 99.3% of participants interviewed, this participant reported never having had malaria. She also reported that 2 of her 3 children had never had malaria. This lack of personal experience with a disease that had otherwise greatly impacted her community might explain her reluctance to accept a TBV.

Given the almost-nil frequency of TBV rejection in the study, it was not possible to directly test the hypothesis that decreased malaria exposure leads to decreased TBV acceptability. Instead three alternate hypotheses were chosen for testing, which may serve as a proxy for the effect of personal malaria exposure on vaccine acceptability: a high degree of malaria exposure will make an individual more likely to (1) pay for a TBV; (2) agree to receive multiple doses of a TBV; and (3) agree to receive a less effective vaccine as compared to someone with little or no personal malaria exposure. For the purposes of this study, malaria exposure was categorized as none (0 prior cases of malaria), low (1–4 prior cases), or high (5 or more cases, including those who reported too many cases to count or remember).

Statistical analysis using Fisher’s exact test revealed no relationship between malaria exposure and willingness to pay (p = 0.92); price willing to pay (p = 0.48); or price range category of none (0 soles), low (1–9 soles), or high (10 or more soles) (p = 0.51). Further, level of prior malaria exposure was not predictive of willingness to receive multiple vaccine doses (p = 0.85) or willingness to receive a 70 or 50% effective vaccine (p = 0.81 and p = 0.79, respectively). All p-values remained statistically insignificant when malaria exposure was further re-categorized as simply high (5 or more cases) or low (4 or fewer cases).

Asked about willingness to receive a transmission-blocking malaria vaccine, participants in all 6 communities gave answers that demonstrated *both* a clear understanding that such a vaccine would not protect the vaccinated individual from becoming infected *and* clear support for receiving a vaccine with those characteristics (Table 3).

**Table 3:** Reasons cited by participants for the acceptability of a transmission-blocking malaria vaccine, demonstrating motives and understanding of important concepts

Theme 1: A TBV might be a superior intervention to current prevention and treatment options
*“It’s necessary for not infecting other people; my daughter has gastritis from getting sick so much!”*—36 year old male, 12 de Abril
*“So that [the mosquitoes] perhaps won’t transmit, for prevention; besides, it’s better than the pills.”*—53 year old male, Santa Rita
*“To not contaminate other people and so that it’s easier to get healthy. You can’t put up with this.”*—26 year old female, 12 de Abril
*“It’s better than the pills that give us allergies.”*—27 year old female, La Habana
*“It would give time, and there would be someone [healthy] left in the house that could still attend to the sick.”*—34 year old female, La Habana
Theme 2: The concept of transmission blockade is understood and acceptable.
*“So that it doesn’t go on increasing, to not infect the rest.”*—29 year old female, San José de Lupuna
*“I wouldn’t want malaria to keep on growing, and this avoids that it passes on to all the others.”*—40 year old female, Santa Rita
*“The mosquito will no longer transmit it; if we don’t, it will keep on infecting.”*—65 year old male, San Pedro
*“I’m protecting it from transmitting to everybody else.”*—39 year old female, Cahuide
*“So that my children don’t get sick and pass it on to all the other children.”*—*2*0 year old female, Cahuide
Theme 3: The altruistic nature of a TBV is not a deterrent
*“We’re avoiding that my partner or a child or grandchild that comes to visit gets sick.”*—75 year old male, Cahuide
*“So that the disease doesn’t pass on, to protect the rest. Perhaps it will be that it doesn’t do me any good, but it’s for others.”*—35 year old male, La Habana
*“For protecting other people, there’s no problem with that.”* 57 year old male, San Pedro
*“So there wouldn’t be so much transmission to other people. I would be protecting my children more than anything.”*—33 year old female, Cahuide
*“To protect everyone else from the illness.”*—38 year old male, Santa Rita
Theme 4: Appropriate application of a TBV could lead to regional elimination of malaria
*“The mosquito won’t transmit to other people anymore; if we all vaccinate ourselves, this malaria won’t exist anymore*—42 year old female, Cahuide
*“The mosquito will no longer transmit to other people, and we won’t have malaria anymore.”*—57 year old female, San José de Lupuna
*“It’s better that we’re not infecting one another anymore. We’d have to vaccinate everyone, then.”*—75 year old male, Santa Rita

Open-ended responses have been categorized by theme

## Discussion

In this study of the acceptability of a herd-immunity-focused, transmission-blocking malaria vaccine that does not directly protect recipients against malaria infection, we found that members of six malaria-hypoendemic communities in the Amazon River basin in Loreto, Peru, overwhelmingly reported such a vaccine to be acceptable for themselves and their children. Although some malaria researchers and policy-makers in the industrialized world have considered a transmission-blocking vaccine (TBV) to be altruistic and, therefore, perhaps less acceptable, the results presented here indicate that affected populations may consider the protection of others a compelling rationale to vaccinate themselves and their children against malaria, especially in light of the prevention and treatment options currently available, primarily LLIN distribution plus opportune diagnosis and treatment. Further, as previously noted, the concept of vaccines aimed at herd immunity to prevent disease transmission in populations is well established [4, 29, 30, 59]. Acceptability of a TBV remained high among the study participants despite characteristics such as having to pay for the vaccine themselves and the potential need for multiple injections.

In 2011, the RTS,S vaccine developed by GlaxoSmithKline (GSK), which targets a sporozoite surface protein, reached phase III trials in sub-Saharan Africa. Modest vaccine efficacy has been documented across multiple clinical trials in adults, children, and infants [60–62]. More recently, safety and tolerance testing has been performed with whole aseptic, purified, cryopreserved Pf sporozoites (PfSPZ) [63–65]. Asexual blood stage vaccine candidates have been proposed since the 1980s, when SPf66 initially gave promising results that were ultimately not replicable [66–68]. Many other asexual blood stage candidates have been proposed and tested with limited success, including MSP1, MSP2, RSEA, and AMA1, stimulating the suggestion of a combined vaccine with both pre-erythrocyte and erythrocyte stage components, as well as a vaccine directed against the whole parasite in the asexual blood stage [69–76].

Given only modest success with current vaccines aimed at the sporozoite and asexual blood stages of malaria parasites, a transmission-blocking vaccine could serve as a key component of malaria control and elimination strategies. Even in low transmission areas, such as Peru, Brazil, and Colombia, published data demonstrate that asymptomatic malaria parasitaemia and gametocytaemia contribute to *Plasmodium* transmission [77–80]. These data suggest that a TBV, even if only moderately effective, would contribute to reducing transmission from individuals with subclinical infection.

This study has several limitations. First, the study participants were selected from an ongoing study cohort of individuals under the auspices of the ICEMR-Amazonía. All members of the study have monthly blood samples taken and analysed for the presence of *Plasmodium* species. Those that are positive are treated free of charge at the local health post. Because of their participation in this study, our respondents probably had a better understanding of malaria transmission, pathogenesis, and current treatment limitations than members of other communities with high levels of malaria infection. Self-reports of malaria history are more likely accurate: because of the ICEMR, the majority of these reports likely derive from parasite-based diagnoses rather than episodes of generalized fever or illness. Such universal reports of infection support the assertion that these and similar communities in the region experience endemic malaria and demonstrate the degree to which the illness weighs on community consciousness [35, 48–50, 58]. It is also possible that the familiarity of these communities with the ICEMR and study team members led to social desirability bias: because of their personal relationships with study team members, survey participants may have been more trusting, more anxious to please the surveyors, and expressed higher levels of altruism than residents of a community with no previous knowledge of the ICEMR or team. Future work could repeat this study design in a different population to assess whether acceptance remains high in the setting of low levels of laboratory-proven exposure to malarial disease and where residents do not have an existing relationship with the study team.

Second, the values of family, community, and selflessness were clearly seen to be of critical importance for the members of these six communities and served as motivations for receiving a transmission-blocking malaria vaccine. It is possible that not every community with endemic malaria holds the value of community in equally high regard. This would likely be reflected in a lower number of people that would elect to receive a vaccine that would not protect them directly but would rather serve to benefit the community as a whole.

Third, this study was carried out in a region where malaria transmission is not intense. Results of a similar survey might be different even in other parts of Peru, such as in the nearby city of Iquitos, where malaria is far less common, and Lima, which is entirely unaffected; survey results could likewise differ in places with higher disease burden, such as in malaria holoendemic regions of sub-Saharan Africa and Papua New Guinea. This could impact success at achieving malaria elimination following the introduction of a TBV. Any one or a combination of these reasons could help explain why participants in the current study expressed greater willingness to accept a TBV than participants of studies elsewhere were to accept a malaria vaccine that would prevent human infection [81, 82].

Given these limitations, this study should be adapted and expanded in communities with varying degrees of malaria exposure. Until such studies are carried out, it would be difficult to make any claim for the generalizability of the results of this study. This would also help us better evaluate a new hypothesis that degree of malaria exposure correlates with TBV acceptability. With only two subjects having no personal experience with malaria, the present study was unable to demonstrate any such relationship, should it exist. It would also be profitable to directly compare the acceptability of transmission-blocking and infection-preventing malaria vaccines in this and other communities, as this was not done in this study. In addition, cultural and educational factors may affect TBV acceptability in other malaria-endemic regions; similar studies in other places should take such factors into account.

Finally, this study did not assess the potential impact of adverse events (AEs) on willingness to receive the TBV. It is widely known that parents and caregivers may refuse to allow their children to be vaccinated for fear of AEs even when such fear is unwarranted [83]. Little is known about the exact nature of the relationship between perceived probability or severity of an AE and vaccine acceptance. Nevertheless, it seems reasonable to assume that acceptance would decrease with increasing frequency and severity of expected AEs, a phenomenon not addressed by this study.

Several studies have commented on community perceptions of and concerns about infection-preventing malaria vaccines [81, 82]. When reviewing the literature, however, it appears that the present study is the first to assess attitudes toward the idea of a transmission-blocking malaria vaccine in a malaria-hypoendemic region. Future studies should include qualitative methods such as in-depth interviews to better understand participant responses to the types of quantitative questions reported here and to explore in greater depth potential facilitators and barriers to vaccine acceptance among this population. Such research will be crucial to effective promotion of an eventual TBV to create anti-malaria herd immunity in populations.

## Additional files

**Additional file 1.** Study questionnaire translated into English.

**Additional file 2.** All responses to survey question #18: why people would or would not be willing to receive or allow their children to be vaccinated with a vaccine that confers no personal protection (a TBV).
