# Supplementary material for: Acceptability of a herd immunity-focused, transmission-blocking malaria vaccine in malaria-endemic communities in the Peruvian Amazon: an exploratory study
Source: Malar J. 2018 Apr 27;17:179. doi: 10.1186/s12936-018-2328-z (PMC5921293; doi:10.1186/s12936-018-2328-z)
Supplement: Supplementary file 1 — Additional file 1. Study questionnaire translated into English. [file 12936_2018_2328_MOESM1_ESM.docx]

**Additional file 1: S1: Study questionnaire translated into English**

1. Sex M/F
2. How old are you?
3. What is your profession?
4. What religion do you practice (if any)?
5. Where do you live?
6. How many children do you have?
7. How old are your children?
8. How many people live in your home? How old are they?
9. What is your level of education? What is the highest grade you completed?
10. Have you received a vaccine against any illness(es)? Which one(s)?
11. Have your children received vaccine(s) against any illness(es)? Which one(s)?
12. Have you ever had malaria? How many times? How long ago was the last time?
13. Have your children ever had malaria? How many times? How long ago was the last time?
14. What do you currently do or use to prevent yourself from getting malaria? Bed net? Baygón or repellent? Clothing? Pills? Others?
15. Would you want to receive a vaccine against malaria?
16. A traditional vaccine keeps you from getting sick. But malaria isn’t a traditional disease because it transmits from person to mosquito and then from that mosquito to another person and not simply from person to person like some other diseases. So imagine that there was a malaria vaccine, but it wouldn’t prevent you from getting malaria. Instead it would prevent that the mosquitoes that bite you from getting malaria and transmitting it to others. In other words, even after getting the vaccine, you could still get sick with malaria, but you would no longer pass it on to others in your community. Would you be willing to receive a vaccine like that?
17. Would you be willing to give a vaccine like that to your children?
18. Why or why not?
19. Up to what price would you pay for a vaccine like that? Only if it were free? Up to 5 soles per person? Up to 10 soles? Up to 20 soles? More? Less?
20. Would you be willing to receive a vaccine like that if it were an injection? If they were drops in the mouth? If it was a pill?
21. Would you be willing to give a vaccine like that to your children if it were an injection? If they were drops in the mouth? If it was a pill?
22. Would you be willing to receive a vaccine like that if it was only one injection? What if you had to return to the health post or hospital to receive multiple injections?
23. Would you be willing to give a vaccine like that to your children if it was only one injection? What if you had to return to the health post or hospital to receive multiple injections?
24. Would you be willing to receive a vaccine like that if it worked 100% of the time? 70% of the time? 50% of the time?
25. Would you be willing to give a vaccine like that to your children if it worked 100% of the time? 70% of the time? 50% of the time?
