# Supplementary material for: Acceptability of a herd immunity-focused, transmission-blocking malaria vaccine in malaria-endemic communities in the Peruvian Amazon: an exploratory study
Source: Malar J. 2018 Apr 27;17:179. doi: 10.1186/s12936-018-2328-z (PMC5921293; doi:10.1186/s12936-018-2328-z)
Supplement: Supplementary file 2 — Additional file 2. All responses to survey question #18: why people would or would not be willing to receive or allow their children to be vaccinated with a vaccine that confers no personal protection (a TBV). [file 12936_2018_2328_MOESM2_ESM.docx]

**Additional file 2: S2: All responses to survey question #18: why people would or would not be willing to receive or allow their children to be vaccinated with a vaccine that confers no personal protection (a TBV)**

| Cahuide |
| --- |
| "So we all stop getting sick. If it’s there, why refuse it?" - 68 year old male |
| "So that we would all be protected." -38 year old female |
| "Who wants to have malaria?" -66 year old female |
| "So there wouldn't be so much transmission to other people. I would be protecting my children more than anything." -33 year old female |
| "It’s against malaria; that’s good." -39 year old female |
| "So that malaria doesn’t harm us." -32 year old female |
| "It’s more feasible than the pills. If it’s for prevention, that's magnificent!" -30 year old female |
| "So we don’t get sick." -64 year old female |
| "Because it protects us against that disease." -19 year old female |
| "To prevent the mosquitoes from infecting us." -76 year old female |
| "To protect ourselves, to not have any more [malaria]." -48 year old female |
| "I’m protecting it from transmitting to everybody else." -39 year old female |
| "The mosquito won’t transmit to other people anymore; if we all vaccinate ourselves, this malaria won’t exist anymore." -42 year old female |
| "So that they [my children] won’t get malaria; a vaccine is better than the pills." -32 year old female |
| "So that I don’t pass it on to my children." -56 year old female |
| "It’s in my own interest that malaria doesn’t keep spreading anymore." -57 year old female |
| "If it has to do with eradicating malaria, who wouldn’t want it? But I don’t think it can be the mosquito." -30 year old male |
| "So that I can have peace of mind." -64 year old female |
| "It wouldn’t be able to infect my children." -47 year old male |
| "A vaccine is better than the pills." -58 year old female |
| "To be healthy, to avoid the disease." -18 year old female |
| "It’s a block for the whole family." -52 year old male |
| "So that they [my children] won’t get sick." -21 year old female |
| "So that I won’t infect all the others anymore." -29 year old male |
| "Because then it won’t pass on anymore." -26 year old female |
| "They [my children] won’t be taking those pills anymore." -25 year old female |
| "It’s necessary for us to be protected." -29 year old female |
| "So that we don’t get sick." -30 year old male |
| "To avoid so much illness, so that they [the children] don’t get sick." -63 year old female |
| "We’re avoiding that my partner or a child or grandchild that comes to visit gets sick." -75 year old male |
| "To no longer transmit malaria." 57 year old male |
| "So that they [my daughters] don’t get sick." -20 year old female |
| "To prevent malaria." -52 year old male |
| "For us all to protect ourselves." -49 year old male |
| "So that my children don't get sick and pass it on to all the other children." -20 year old female |
| "So my children don’t get sick, so as not to transmit it." -29 year old female |
| "To prevent transmission." -40 year old male |
| "It would be better than the pills." -52 year old female |
| "To prevent sickness." -76 year old male |
| "So that we all no longer get sick from malaria." -44 year old female |
| "To protect ourselves." -34 year old female |
| "I would be protecting." -23 year old male |
| "We could all be protected." -48 year old female |
| "If you give the vaccine to everyone, then they won’t get malaria." -51 year old female |
| "Because it’s protecting us from malaria." -49 year old male |
| 12 de Abril |
| "There are so many illnesses with malaria." -62 year old female |
| "We would like to no longer get sick from malaria here." -61 year old female |
| "To not transmit it to the whole house and to others." -39 year old female |
| "We need it, we need a cure." -52 year old male |
| "I don’t want to see them [my children] sick, this malaria is very dangerous." -30 year old female |
| "If it protects my family from malaria, [that's good]." -42 year old male |
| "To avoid infecting other people." -48 year old male |
| "To protect against malaria." -43 year old male |
| "Because then it doesn’t transmit to another person." -27 year old male |
| "To no longer transmit malaria." -33 year old female |
| "To not contaminate other people and so that it's easier to get healthy. You can't put up with this." -26 year old female |
| "To prevent them [sic], to not be infecting others." -78 year old female |
| "To prevent my children from getting sick." -78 year old male |
| "It’s necessary for not infecting other people; my daughter has gastritis from getting sick so much!" -36 year old male |
| "To not pass it on; I want everyone to be healthy." -47 year old female |
| "It’s necessary to help." -75 year old male |
| "To prevent them [my children] from getting sick." -34 year old male |
| San José de Lupuna |
| "Better than the pill." -68 year old male |
| "Why not, if it’s for health?" -59 year old male |
| "For all to be protected." -39 year old female |
| "So malaria no longer gets transmitted." -49 year old female |
| "It’s better that way." -26 year old female |
| "So that we don’t get sick from malaria." -48 year old female |
| "So that other people don’t get sick." -40 year old female |
| "So that it doesn’t go on increasing, to not infect the rest." -29 year old female |
| "The mosquito will no longer transmit to other people, and we won’t have malaria anymore." -57 year old male |
| "We want to be healthy, we don’t want to have this disease anymore." -59 year old female |
| "If it’s a remedy, why not?" -71 year old male |
| "It’s so necessary, malaria is painful." -75 year old male |
| "To avoid infecting other people." -66 year old male |
| "I don’t want my daughters to have malaria." -45 year old female |
| "It’s for the family; the disease doesn’t give warning." -69 year old male |
| "They [my children] would be protected from malaria." -29 year old female |
| "Many won’t suffer from it anymore." -61 year old male |
| "To protect." -35 year old male |
| "So that they [my children] don’t get sick." -60 year old female |
| "I would prefer to prevent my children from getting sick." -33 year old female |
| "To prevent malaria." -19 year old female |
| "So that I can protect them [my children] from malaria." -33 year old female |
| "To protect my children from malaria." -39 year old female |
| "So they [my children] don’t get so sick from malaria, as we are now." -38 year old female |
| "For the prevention of malaria." -20 year old male |
| "To protect against malaria." -37 year old female |
| "To not transmit to others." -26 year old female |
| San Pedro |
| "So that they are protected." -70 year old male |
| "Better than taking the pills." -35 year old female |
| "It’s going to protect a little bit against malaria." -27 year old female |
| "For protecting other people, there’s no problem with that." -57 year old male |
| "To not keep transmitting it to everyone else." -41 year old female |
| "To no longer infect." -63 year old male |
| "To protect ourselves from that disease." -30 year old female |
| "So that we no longer have this malaria." -30 year old female |
| "The mosquito will no longer transmit it; if we don’t, it will keep on infecting." -65 year old male |
| "To hold back this malaria, to no longer transmit." -57 year old female |
| "To not give it to others anymore, not as much." -43 year old female |
| "We get it all the time, and we want to be healthy." -36 year old female |
| "To not transmit it to others anymore." -50 year old female |
| Santa Rita |
| "If it’s for prevention." -40 year old female |
| "To avoid infecting." -33 year old female |
| "It’s going to protect you from the mosquito; they would have a cap." -44 year old female |
| "It's better that we're not infecting one another anymore. We'd have to vaccinate everyone, then." -75 year old male |
| "If there is a vaccine for prevention, who is not going to want it? It’s a hope that can sustain you." -48 year old male |
| "Why not? Because we all get malaria." -76 year old male |
| "So that they can live healthy." -69 year old female |
| "We will not infect everyone else." -50 year old female |
| "If it’s trying to treat, why not?" -73 year old male |
| "It has to be, it’s like a treatment." -49 year old male |
| "It would be more preventable." -27 year old female |
| "Because it would protect us." -48 year old female |
| "We have to prevent it, neither are we going to live like animals." -39 year old male |
| "More would be protected from malaria." -22 year old female |
| "Who will not want it? We are sick so often because of malaria." -34 year old female |
| "I wouldn’t want malaria to keep on growing, and this avoids that it passes on to all the others." -40 year old female |
| "It would be good for them [my children], to protect them." -33 year old female |
| "To prevent malaria." -55 year old male |
| "So that [the mosquitoes] perhaps won’t transmit, for prevention; besides, it’s better than the pills." -53 year old male |
| "To prevent them [my children] from having that disease." -42 year old female |
| "To protect everyone else from the illness." -38 year old male |
| La Habana |
| "So that the disease doesn’t pass on, to protect the rest. Perhaps it will be that it doesn’t do me any good, but it’s for others." -35 year old male |
| "If it’s there, why not? It would be sent from God to protect us in that way." -68 year old male |
| "So that we don’t get sick anymore." -44 year old male |
| "Many mosquitoes bite me. If it’s for protection, why refuse it? It would be good for our health." -56 year old male |
| "Supposedly it would be an excellent vaccine, and I would avoid getting sick anymore, even if not directly." -62 year old male |
| "To protect our bodies." -46 year old male |
| "So that my children can’t get sick." -25 year old female |
| "The pills cause a lot of harm." -30 year old male |
| "To protect my children, so that they don’t get that disease." -33 year old female |
| "It’s better than the pills that cause allergies." -27 year old female |
| "It would give us time, so that there would be someone who could take care of the sick person." -34 year old female |
| "To no longer infect the rest of my children." -64 year old female |
| "Better than having to take pills." -26 year old female |
